# A Comparison of Denoising Approaches for Spoken Word Production Related Artefacts in Continuous Multiband fMRI Data

**DOI:** 10.1162/nol_a_00151

**Published:** 2024-09-11

**Authors:** Angelique Volfart, Katie L. McMahon, Greig I. de Zubicaray

**Affiliations:** Faculty of Health, School of Psychology and Counselling, Queensland University of Technology, Brisbane, Australia; Faculty of Health, School of Clinical Sciences, Queensland University of Technology, Brisbane, Australia; Herston Imaging Research Facility, Royal Brisbane & Women’s Hospital, Brisbane, Australia; Centre for Biomedical Technologies, Queensland University of Technology, Brisbane, Australia

**Keywords:** functional magnetic resonance imaging, multiband echoplanar imaging, naming to definitions, spoken word production

## Abstract

It is well-established from fMRI experiments employing gradient echo echo-planar imaging (EPI) sequences that overt speech production introduces signal artefacts compromising accurate detection of task-related responses. Both design and post-processing (denoising) techniques have been proposed and implemented over the years to mitigate the various noise sources. Recently, fMRI studies of speech production have begun to adopt multiband EPI sequences that offer better signal-to-noise ratio (SNR) and temporal resolution allowing adequate sampling of physiological noise sources (e.g., respiration, cardiovascular effects) and reduced scanner acoustic noise. However, these new sequences may also introduce additional noise sources. In this study, we demonstrate the impact of applying several noise-estimation and removal approaches to continuous multiband fMRI data acquired during a naming-to-definition task, including rigid body motion regression and outlier censoring, principal component analysis for removal of cerebrospinal fluid (CSF)/edge-related noise components, and global fMRI signal regression (using two different approaches) compared to a baseline of realignment and unwarping alone. Our results show the strongest and most spatially extensive sources of physiological noise are the global signal fluctuations arising from respiration and muscle action and CSF/edge-related noise components, with residual rigid body motion contributing relatively little variance. Interestingly, denoising approaches tended to reduce and enhance task-related BOLD signal increases and decreases, respectively. Global signal regression using a voxel-wise linear model of the global signal estimated from unmasked data resulted in dramatic improvements in temporal SNR. Overall, these findings show the benefits of combining continuous multiband EPI sequences and denoising approaches to investigate the neurobiology of speech production.

## INTRODUCTION

From the late 1990s, multiple functional magnetic resonance imaging (fMRI) studies demonstrated that producing speech introduces blood oxygen level dependent (BOLD) signal artefacts in continuous single-band gradient echo echo-planar imaging (EPI) acquisitions (e.g., Barch et al., 1999; Birn et al., 1998; Gracco et al., 2005; Mehta et al., 2006; Palmer et al., 2001). These artefacts are typically located over basal areas, areas adjacent to cerebrospinal fluid (CSF)/brain edges and lateral frontal cortical areas (Mehta et al., 2006). As they are task-correlated sources of variability, failure to mitigate/remove them compromises the accurate detection of brain regions involved in speech production.

Rigid body motion (i.e., bulk head movement), cardiac cycle pulsatile motion of CSF/tissue boundaries (referred to as CSF/edge effects, i.e., signals arising from voxels comprising purely CSF as well as those voxels on CSF/tissue boundaries that involve partial voluming artefacts), and respiration related signal changes have all been shown to be significant sources of local signal fluctuations and magnetic field distortions for fMRI studies, whether they be task-based or resting-state studies (e.g., Power et al., 2012, 2014). These noise sources are exacerbated when speech is produced, due to changes in respiration (inhalation, exhalation, and pauses) and movements of the tongue and facial muscles that occur prior to, during, and following articulation (Birn et al., 1998; Gracco et al., 2005; Mehta et al., 2006). Fluctuations in the whole brain (i.e., global) signal are also strongly influenced by these variables, especially respiration (Power et al., 2018; Power, Plitt, Laumann, et al., 2017). While respiratory variations in resting-state fMRI global signal typically manifest as slow and regular large amplitude changes (see Power et al., 2018), speech-related respiration changes manifest as significant decreases and increases in global signal intensity during and several seconds following the speech envelope, respectively (Mehta et al., 2006). Extracranial signal changes observable in the vicinity of the temporalis muscle bilaterally are also correlated with global signal intensity during the speech envelope and are associated with blurring of the BOLD signal (i.e., partial volume artefacts) over the inferior lateral frontal cortex (Mehta et al., 2006; Palmer et al., 2001).

Not surprisingly, both experimental design and post-processing (denoising) techniques have been proposed and implemented over the years to mitigate/remove the various noise sources. At the design level, sparse or compressed EPI sequences capitalise on the delay in the task-related BOLD signal response to acquire temporally segregated noncontinuous images that are relatively free of spoken word production motion-induced artefacts and scanner acoustic noise, providing comparable sensitivity to continuous fMRI acquisitions as shown with nonspeech tasks (e.g., de Zubicaray et al., 2001; Gracco et al., 2005; Nebel et al., 2005). As early fMRI studies demonstrated that residual motion related signal changes persist even after post-processing with rigid body realignment algorithms (e.g., Grootoonk et al., 2000), production researchers have often included speech envelopes (onset to offset) and movement parameters as nuisance regressors in their analyses and/or implemented realign and unwarp algorithms that attempt to model and remove susceptibility-by-movement interactions in continuous data (e.g., Ekert et al., 2021; Wilson et al., 2009). In cases of more severe movement detected by realignment algorithms, scrubbing/censoring of outlier images has also been employed (Xu et al., 2014). While realignment algorithms can be applied to continuous fMRI data either before or after slice-timing correction, it is important to note that motion and scrubbing estimates calculated following temporal interpolation do not accurately reflect the extent of movement in a time series and using them in subsequent post-processing or analysis stages will compromise the accurate detection of task-related activity (Power, Plitt, Kundu, et al., 2017).

Component-based post-processing or denoising techniques have also been applied to estimate and remove sources of motion and physiological noise from continuous fMRI acquisitions (e.g., see Caballero-Gaudes & Reynolds, 2017). Most, such as aCompCor (Behzadi et al., 2007) or ICA-AROMA (Pruim et al., 2015), employ principal or independent component analysis (PCA and ICA, respectively) to separate noise sources from the signal of interest. The former derives noise components from CSF and white matter (WM) maps determined from segmented anatomical images, while the latter uses a set of four predefined temporal and spatial features. Both techniques have been applied in studies using continuously acquired fMRI production data (e.g., Janssen & Mendieta, 2020; Krishnamurthy et al., 2021; Todorović et al., 2024). While both approaches decompose the BOLD signal time course into task- versus noise-related variance, ICA identifies statistically independent subcomponents that may not be orthogonal and PCA maximizes variance in orthogonal components. The accuracy of these techniques is affected both by the use of long repetition times (TR; >2 s) in single band EPI acquisitions, which prevents adequate sampling of cardiac and respiratory frequencies (<1 Hz), and by decisions regarding which components to remove in order to preserve signal of interest (e.g., see Caballero-Gaudes & Reynolds, 2017). It is well known that CSF/edge signal intensity variations reflect cardiac and respiratory cycles that are directly influenced by speech (e.g., Farthing et al., 2007; Mehta et al., 2006; Wang et al., 2022). However, multiple studies have demonstrated task-related BOLD responses in WM, indicating these signals should not routinely be treated as nuisance regressors as this will also remove effects of interest (Gawryluk et al., 2014; Grajauskas et al., 2019; Schilling et al., 2023).

Finally, global signal regression (GSR) is an additional post-processing step that can be used to model and remove respiratory and muscle related variability that component-based techniques are unable to adequately remove (Liu et al., 2017; Power et al., 2018). However, approaches to GSR vary. Global signal can be calculated per-volume from masked or unmasked data and then removed either prior to or during analysis as a regressor of no interest. For example, the approach implemented in the CONN toolbox (Nieto-Castañón & Whitfield-Gabrieli, 2022) involves calculating the change in average BOLD signal within masked data, that is, excluding extracranial sources, which is then entered as a covariate of no-interest in the voxel-level general linear model analysis. Alternatively, the approach implemented by Macey et al. (2004) removes global effects estimated from unmasked data in a separate step, with the residual time course data then entered into analysis. While GSR is a well-established technique in resting-state fMRI studies, the topic has received relatively little discussion in the speech production literature. The task-correlated nature of global signal fluctuations that occur during and following the speech envelope (e.g., Mehta et al., 2006) also raises questions about when it might be appropriate to employ GSR. For example, if the study goal is to investigate aspects of speech motor control, then it could be argued that GSR should probably not be employed as it will likely also remove non-global signals associated with articulatory mechanisms. Conversely, if the goal is to investigate processes preceding articulation, then GSR might confer benefits by removing noise attributable to the respiratory and muscle activity associated with the act of speaking.

### Multiband EPI Acquisitions

In recent years, simultaneous multislice (SMS) or multiband (MB) EPI sequences have been increasingly adopted in fMRI studies of spoken word and narrative production, with the majority employing continuous (e.g., Morales et al., 2022; Roos et al., 2023; Todorović et al., 2024) rather than sparse sampling acquisitions (e.g., Correia et al., 2020). These sequences offer better signal-to-noise ratio (SNR) and temporal resolution, allowing for adequate sampling of respiration and cardiovascular signals and reduced scanner acoustic noise. The additional volumes acquired with shorter TRs also improve statistical power compared to single band acquisitions (Wall, 2023). However, these new sequences may also introduce additional artefacts. For example, high slice acceleration factors (i.e., >4), small voxel sizes (<2 mm), and very short TRs (<1 s) result in exponentially lower SNR and poorer contrast that reduces the efficiency of motion realignment and registration algorithms and can introduce false-positive activation due to signal leakage between slices (McNabb et al., 2020; Todd et al., 2016; see also Wall, 2023). Multi-echo (ME) MB EPI sequences that acquire several images at different echo times (TEs) allow separation of non-BOLD (e.g., rigid body motion) artefacts from BOLD responses and show better sensitivity to signal changes in regions affected by magnetic field distortions, yet require longer TRs (>1 s) that may not adequately sample cardiac and respiratory frequencies (<1 Hz) and additional post-processing steps (e.g., DuPre et al., 2021; Kundu et al., 2017).

### The Present Study

Despite the increased use of continuous MB EPI sequences in speech production fMRI studies, to our knowledge there are currently no studies that have characterized the associated noise sources and the effects of removing them. This seems somewhat incongruous given the extensive discussion of these topics for resting-state fMRI datasets that have less severe artefacts to contend with. In the present study, we employed a continuous MB EPI sequence to acquire fMRI data while healthy participants performed an auditory naming to definition task entailing delayed responding. After applying a typical fMRI pre-processing pipeline consisting of realignment and unwarping, spatial normalisation, smoothing, and regressors for the relevant event types, we first examine the variance attributable to each of the abovementioned noise sources and in combination: residual and outlier motion, CSF/edge effects, and global signal. In addition, we compare the two different approaches to GSR. Next, we show the effects on task-related (definition vs. control) activation when these sources of noise are/are not included as nuisance regressors.

## MATERIALS AND METHODS

### Participants

We recruited 18 healthy right-handed native English speakers (11 females; mean age = 23.86 ± 5.83) among students and staff of Queensland University of Technology (QUT). All participants reported normal or corrected-to-normal vision, no hearing deficits, no history of neurological or psychiatric disorder or learning disability or use of psychotropic medication (e.g., antidepressants). All participants gave written consent to participate in the experiment according to the protocol approved by Metro North Health (HREC/15/QRBW/447) and QUT (2015-1148-1620) Human Research Ethics Committees and were compensated with AUD30 for their involvement in the study.

### Stimuli

We selected 50 auditory definitions from previously validated tests: 15 were taken from Hamberger and Seidel (2003), 13 were taken from Hamberger et al. (2022), and 22 were taken from Yochim et al. (2015). Four definitions were also selected to be used as practice stimuli (two from Hamberger et al., 2022, and two from Yochim et al., 2015).

Definitions were recorded by a native Australian English female speaker. They were then processed in Audacity (https://www.audacityteam.org/) for normalization, noise reduction, and volume equalization.

In addition, we also created 50 control stimuli in Audacity by reversing the audio track of each definition to preserve physical features of the item but suppress its meaning. Two of the example definitions were also transformed with the same method to create control practice stimuli.

### Procedure

**Figure 1.**
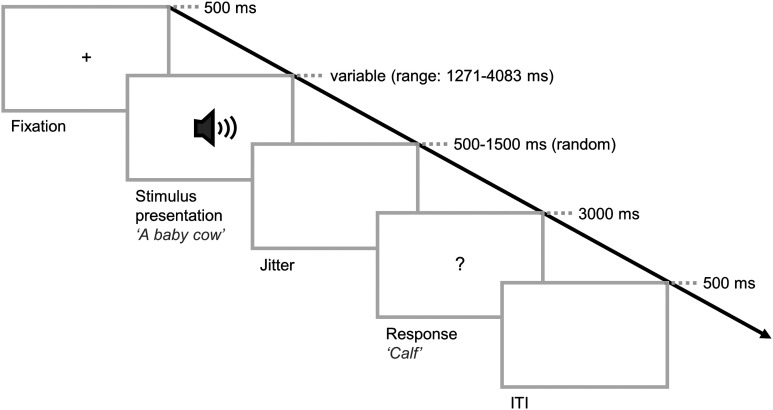
Experimental paradigm performed in the scanner. ITI = intertrial interval.

Each trial followed the same structure (Figure 1): A fixation cross first appeared on the screen for 1,500 ms followed by the sound presentation (range = 1,271–4,083 ms) concomitant with a visual presentation cue (a speaker icon). After a variable delay jittered between 500 and 1,500 ms, a visual response cue (“?”) appeared on the screen for 3,000 ms, indicating that the participant could start naming. A blank interstimulus screen was presented for 500 ms before starting the next trial. The participant’s task was to carefully listen to the auditory stimulus (definition or control) and to name the item with a single word as quickly as possible upon appearance of the visual cue. Participants were asked to produce the word “noise” upon occurrence of control stimuli.

Note that the naming to definition task described here was conducted as part of a larger investigation that also included a naming of environmental sounds task (the results of which to be described elsewhere). The order of presentation of the two tasks was counterbalanced across participants.

After the six practice stimuli, participants were presented with two blocks of 50 trials each with a brief break in-between. Order of items was pseudo-randomized for each participant such that no more than two consecutive stimuli from the same condition (definition or control) were presented.

### Apparatus

The experiment was conducted on a PC and programmed in PsychoPy (Version 2022.2.5; https://www.psychopy.org/; Peirce et al., 2022) for the presentation of stimuli. Auditory stimuli were played to the participant via MRI-compatible headphones (Optoacoustics Active II, Optoacoustics Ltd, Israel) and visual cues were back-projected using a BOLDScreen 32 LCD (Cambridge Research Systems Ltd, UK) projector onto a screen visible by the participant through a mirror attached to the head coil. The participant’s verbal responses were recorded on digital audio files (monophonic audio, 48000 Hz) with a fibre-optic dual-channel noise-cancelling microphone (fOMRI III, Optoacoustics) attached to the head coil (https://www.optoacoustics.com/).

### Analysis of Behavioural Responses

Each participant’s naming response was transcribed and scored for accuracy. The audio files containing participants’ responses were post-processed in Audacity to remove scanner noise and amplify the verbal production when necessary. Naming latencies were then automatically detected using Chronset (https://www.bcbl.eu/databases/chronset; Roux et al., 2017) and further manually checked using a custom script (van Scherpenberg et al., 2020) running in Praat (Version 6.2.14; https://www.fon.hum.uva.nl/praat/; Boersma & Weenik, 2017).

### MRI Acquisition

Neuroimaging data were acquired at the Herston Imaging Research Facility on a 3-Tesla Siemens Magnetom Prisma MRI scanner (Siemens Healthineers, Erlangen, Germany) with a 64-channel head coil. Structural images were collected with a high-resolution 3D T1-weighted image (MPRAGE; TR = 1,900 ms, TE = 2.32 ms, inversion time [TI] = 900 ms, flip angle (FA) = 9°, matrix = 256 × 256, slices = 0.9 mm × 192, field of view [FOV] = 240 × 240). Functional images were collected with an MB twice refocussed EPI sequence (TR = 1,000 ms, TE = 30 ms, FA = 80°, matrix = 72 × 72, slices = 2.6 mm × 48, FOV = 190 × 190 mm^2^, MB acceleration factor = 4, bandwidth 2572 Hz/Px). Each run (1 per experimental block) lasted for 7:49 min, including 10 s of dummy scans at the beginning. At the end of the session, field maps were collected (TR = 520 ms, TE 1 = 4.92 ms, TE 2 = 7.38 ms, FA = 60°, matrix = 72 × 72, slices = 2.6 mm × 50, FOV = 190 × 190 mm^2^) for the purpose of creating voxel displacement maps (VDM). A T2-weighted image was also acquired (TR = 6,300 ms, TE = 100 ms, FA = 150°, matrix = 512 × 512, slices = 4.0 mm × 30, FOV = 220 × 220 mm^2^) with the aim of discarding potential incidental findings.

### Image Analysis

Preprocessing and statistical analyses were conducted using SPM12 (https://www.fil.ion.ucl.ac.uk/spm/software/spm12/) and the CONN toolbox (Version 22.a; Nieto-Castañón & Whitfield-Gabrieli, 2022) in MATLAB R2019B (MathWorks, 2019). First, a VDM was created in SPM12 from the field maps. Next, in CONN, functional data were realigned using the SPM realign & unwarp procedure (Andersson et al., 2001) integrating the VDMs to simultaneously correct for motion, magnetic susceptibility geometric distortions, and their interaction. Potential outlier scans were identified using ART (Whitfield-Gabrieli et al., 2011), defined as acquisitions with framewise displacement above 0.9 mm or global BOLD signal changes above 5 standard deviations (Nieto-Castañón, 2022; Power et al., 2014), and a reference mean BOLD image was computed for each subject by averaging all scans excluding outliers. Functional and anatomical data were coregistered and normalized into standard MNI space, segmented into grey matter, WM, and CSF tissue classes, and resampled to 2 mm isotropic voxels following an indirect normalization procedure (Calhoun et al., 2017; Nieto-Castañón, 2022) using the SPM unified segmentation and normalization algorithm (Ashburner, 2007; Ashburner & Friston, 2005) with the default IXI-549 tissue probability map template. Last, functional data were spatially smoothed with a Gaussian kernel of 8 mm full width half maximum (FWHM). Noise components characterized by six rigid-body realignment parameters (RP), outlier scans (scrubbing parameter), CSF time series (the first 5 aCompCor components) and BOLD global signal changes (in *z*-score units) were extracted. In an additional step, we implemented GSR that takes into account regional variation in global effects and detrending via a voxel-level linear model (voxel-level linear model of the global signal, or LMGS; Macey et al., 2004; https://github.com/paulmmacey/lmgs/tree/master).

Participant- and group-level analyses were then carried out in SPM12. At the first level, event types corresponding to the presentation of each auditory stimulus (definition and control), response cue, and the participant’s verbal output (speech onset/envelope) were modelled. The variance due to each of the noise sources in isolation and in combination was also modelled according to eight different analysis pipelines.

1. **Baseline**: realigned and unwarped, normalized and smoothed data only.
2. **Regression of residual motion (RP) and scrubbing alone**: the six rigid-body realignment parameters and outlier scans were added as nuisance covariates to the design matrix for Pipeline 1.
3. **Regression of CSF/edge effects alone**: the 5 CSF/edge aCompCor noise components were added as nuisance covariates to the design matrix for Pipeline 1.
4. **Regression of global signal changes alone**: the CONN estimated BOLD global signal changes were added as nuisance covariate to the design matrix for Pipeline 1.
5. **Regression of residual motion and edge effects**: combination of noise nuisance covariates used in Pipelines 2 and 3.
6. **Regression of residual motion, edge effects and global signal changes**: combination of all noise nuisance covariates used in Pipelines 2, 3, and 4.
7. **GSR alone**: LMGS (Macey et al., 2004) GSR was applied to the data from Pipeline 1.
8. **Residual motion, CSF/edge effects and LMGS**: RP/scrubbing and 5 CSF/edge aCompCor noise components were entered as nuisance covariates in the design matrix using the LMGS GSR corrected data from Pipeline 7.

We also repeated the analysis in Pipeline 6 without brain masking in order to visualize extracranial sources of noise such as muscle activity (e.g., Mehta et al., 2006). Note that SPM12 uses a default proportional threshold value set at 0.8 to ensure the general linear model only estimates data from voxels whose mean value is at least 80% of the global signal, because the BOLD signal in the brain is higher than that outside of the brain. This is also the mask used by CONN for estimating BOLD global signal. We therefore repeated this analysis with the masking threshold set at 0%.

At the second level, contrast images from each pipeline were entered into one sample *t* tests. Our initial analyses examined the BOLD activity associated with each individual source of noise in isolation (residual motion, CSF/edge effects, or global signal changes) while controlling for the other sources to identify the unique variance associated with each (Pipeline 6). Our next set of analyses investigated the effect of including the noise sources as nuisance regressors on the task contrast of naming definitions vs. naming control stimuli (Definition > Control and its inverse; Pipelines 1–8). All analyses were conducted at the whole-brain level, and we used a height threshold of *p* < 0.001 in conjunction with a spatial cluster extent threshold of *p* < 0.05 (family-wise error [FWE] corrected). Location of peak clusters was determined using the Brainnetome atlas (Fan et al., 2016; https://atlas.brainnetome.org/).

We also investigated the effect of implementing GSR via LMGS on temporal SNR (tSNR; Liu, 2016) using the spmup toolbox (Pernet, 2021; https://github.com/CPernet/spmup) by comparing the time series data from Pipelines 1 and 7. We created a mean tSNR image from each of the two runs with/without GSR and entered them into a paired *t* test in SPM12. Investigation of tSNR for Pipeline 7 was driven by the results.

## RESULTS

### BOLD Signal Changes Due to Individual Noise Sources

**Figure 2.**
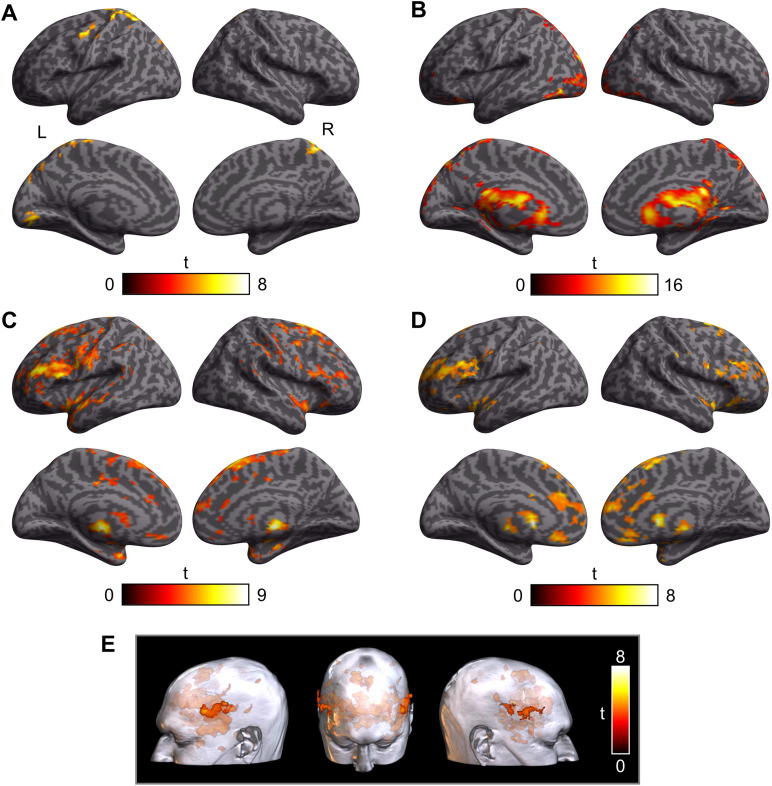
Group-level significant BOLD signal increases. **A**. Clusters showing significant BOLD signal increases due to residual head motion regressors (realignment parameters and scrubbed volumes). **B**. Clusters showing significant BOLD signal increases associated with CSF/edge effects (5 aCompCor components). **C**. Clusters showing significant global BOLD signal increases (CONN method; default mask value set at 80%). **D**. Clusters showing significant global BOLD signal increases (mask value set at 0%). **E**. Extracranial sources of significant global BOLD signal increases observed in the unmasked data from panel D, rendered on a single individual’s T1-weighted MRI scan (‘chris_t1’ in MRIcroGL, Version 13.6.1, https://www.nitrc.org/projects/mricrogl/; Rorden, 2017). A–D are shown on inflated surface renderings from SPM12. All results come from Pipeline 6 looking at the variance coming from each type of noise regressor when controlling for the others and are thresholded at *p* < 0.001 with a spatial extent cluster at *p* < 0.05 (FWE corrected).

**Figure 3.**
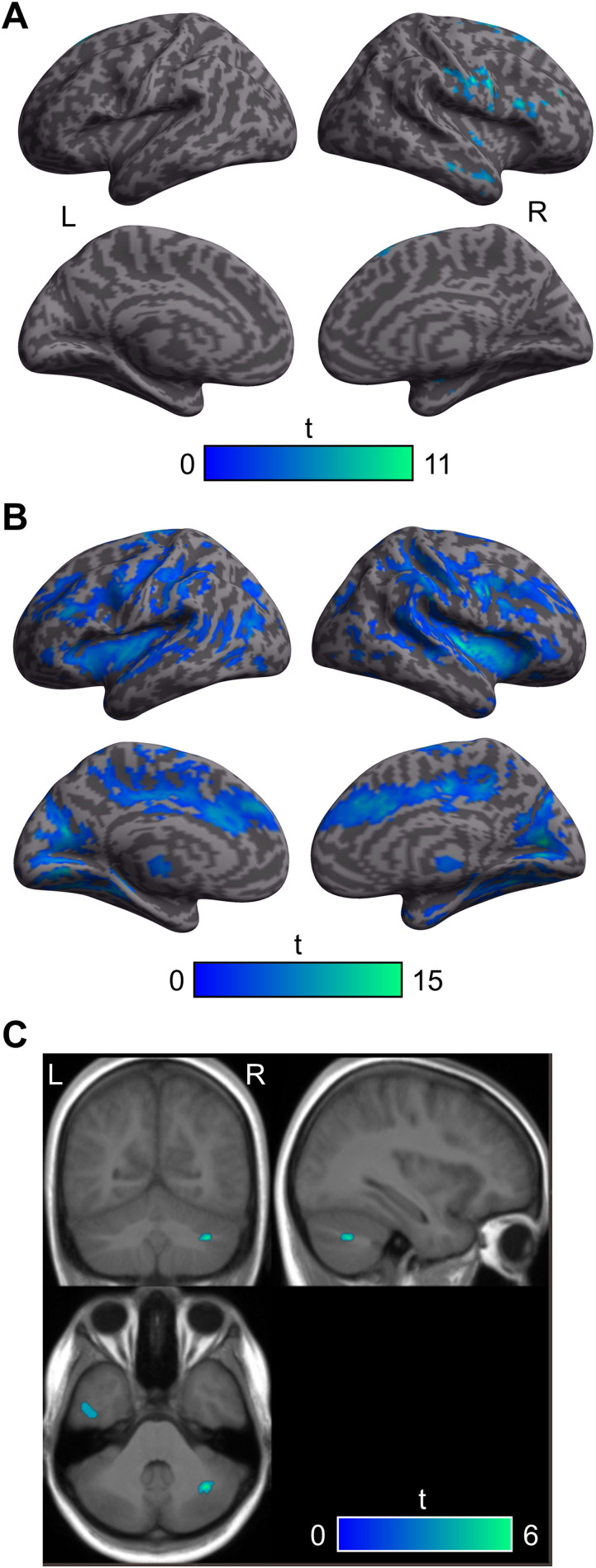
Group-level significant BOLD signal decreases. **A**. Clusters showing significant BOLD signal decreases due to residual head motion (realignment parameters and scrubbed volumes). **B**. Clusters showing significant BOLD signal decreases associated with CSF/edge effects (5 aCompCor components). A and B are shown on a rendered inflated cortical surface from SPM12. **C**. Clusters showing significant global BOLD signal decreases (mask value set at 0%, no significant activity was observed with mask value set at 80%). Clusters are shown on the averaged T1-weighted scan of all 18 participants, and the section view is centred on the peak cluster. All results come from Pipeline 6 looking at the variance coming from each type of noise regressor when controlling for the others and are thresholded at *p* < 0.001 with a spatial extent cluster at *p* < 0.05 (FWE corrected).

Group-level significant BOLD signal increases and decreases due to each individual source of noise (residual motion, CSF/edge effects, and global signal changes) are displayed in Figure 2 and Figure 3, respectively (see also Supplementary Table S1 in the Supporting Information, available at https://doi.org/10.1161/nol_a_00151).

The number of censored/scrubbed volumes across participants and functional runs was variable (mean percentage = 3.2 ± 5.2%), ranging from 0 to 159 (mean = 28.5 ± 45.9, median = 6, mode = 0), with the majority of participants (12/18) having 10 or fewer volumes scrubbed. Increased BOLD signal attributable to residual head motion was observable in five clusters located over the left postcentral gyrus, the left precentral gyrus, the right superior parietal lobule, the left lingual gyrus, and the left superior occipital gyrus (Figure 2A; Table S1). Reduced BOLD signal was also observed in five clusters in the right middle frontal gyrus (MFG), the right superior frontal gyrus (2 clusters, one dorsolateral and one medial), the right putamen, and the right middle temporal gyrus (Figure 3A; Table S1). Increased BOLD signal associated with CSF/edge effects was observed in four large clusters that extended over all areas located in CSF and along its boundaries, especially the ventricles (Figure 2B; Table S1). Decreased BOLD signal was observed bilaterally in four significant clusters with peaks in the right precentral gyrus (one dorsal and one ventral cluster), the right fusiform gyrus, and the brain stem (Figure 3B; Table S1).

Increased global BOLD signal changes were observed in 18 clusters when setting the mask value at the default of 80% (Figure 2C; Table S1) and in 27 clusters when setting the mask value at 0% (Figure 2D–E). These clusters mostly encompassed anterior lateral and medial regions. Interestingly, a large proportion of this activation was located outside the brain, in the vicinity of the temporalis muscle and partial volume artefacts, consistent with Mehta et al.’s (2006) results for gradient echo EPI (Figure 2E). Reduced global BOLD signal (unmasked) was also observed in four significant clusters peaking in the right Crus I of the cerebellum, the left inferior temporal gyrus, and outside the brain (2 clusters; Figure 3C). Note that no significant global BOLD signal decreases were detected when default masking was used.

Importantly, each source of noise (rigid head motion, CSF/edge effects, and global signal) was significant when other noise sources were included as nuisance regressors, indicating they should all be taken into consideration when controlling for speech-related noise.

### Effects of Different Noise Removal Pipelines on Task Contrast Activity (Definition vs. Control)

**Figure 4.**
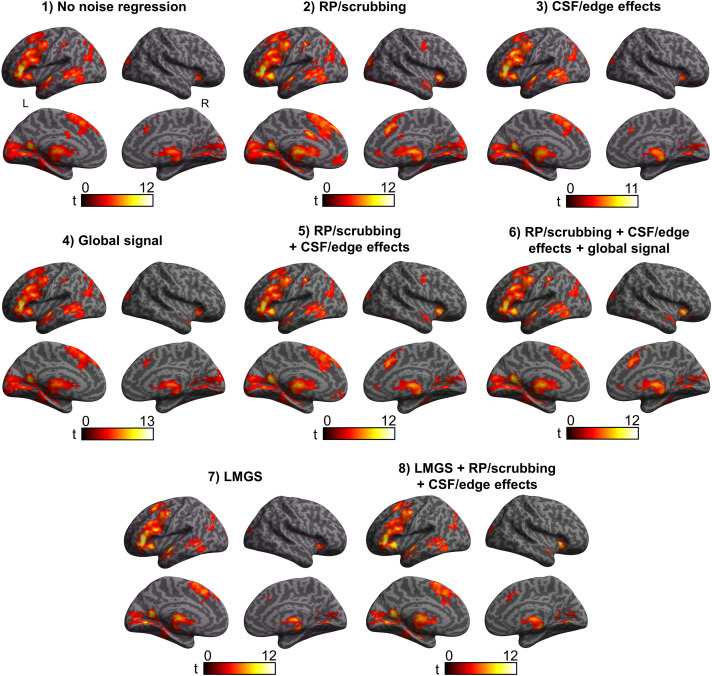
Cortical surface renderings showing significant BOLD signal changes for the contrast Definition > Control as a function of analysis pipeline. All results thresholded at *p* < 0.001 with a spatial extent cluster at *p* < 0.05 (FWE corrected). RP = realignment parameters, CSF = cerebrospinal fluid, LMGS = linear model of the global signal.

While the general location of BOLD signal increase clusters was very similar across the different analysis pipelines (Figure 4), the overall peak maxima (i.e., voxel with maximum *Z* score) and/or size of the peak cluster and its MNI (Montreal Neurological Institute) coordinates exhibited substantial variability (e.g., peak maxima shifting to the right hemisphere with Pipelines 5 and 6, fifteenfold decrease in number of voxels in largest cluster between Pipelines 2 and 8; Table 1).

**Table 1.** Number of clusters with significant BOLD signal and characteristics of the peak *Z*-score cluster for the contrast Definition > Control as a function of noise removal analysis pipeline

Analysis pipeline	Number of significant clusters	Size of peak cluster	*Z* of peak cluster	Coordinates of peak maxima			Peak *Z*-score location
1. No noise regression	5	25,423	6.26	−50	28	2	Left rostral IFG
2. RP/scrubbing	5	35,939	6.27	−50	28	2	Left rostral IFG
3. CSF/edge effects	4	17,607	5.99	−50	28	2	Left rostral IFG
4. CONN global signal	5	28,043	6.40	−44	32	6	Left inferior frontal sulcus
5. RP/scrubbing + CSF/edge effects	8	24,564	6.14	30	28	−2	Right lateral orbital gyrus
6. RP/scrubbing + CSF/edge effects + CONN global signal	7	23,735	6.11	30	26	−2	Right lateral orbital gyrus
7. LMGS	6	4,033	6.29	−6	−60	6	Left parietooccipital sulcus
8. LMGS + RP/scrubbing + CSF/edge effects	7	2,405	6.28	−6	−58	8	Left parietooccipital sulcus

*Note*. IFG = inferior frontal gyrus, RP = realignment parameters, CSF = cerebrospinal fluid, CONN = CONN toolbox, LMGS = linear model of the global signal.

In the absence of noise regression (Pipeline 1), increased BOLD activity was observed in five clusters peaking in the left inferior frontal gyrus (IFG pars triangularis), the right cerebellum, the WM extending subcortically in the right parietal lobe, and the left inferior parietal lobule (IPL; 2 clusters, caudal and rostrodorsal).

After controlling for residual head motion alone (Pipeline 2), all these clusters remained significant except for the WM activation. A new cluster was also observed in the right superior temporal gyrus (STG). After controlling for CSF/edge effects alone (Pipeline 3), the left IFG cluster and one of the IPL clusters (caudal) remained significant with two new clusters now observed in the left anterior STG and right cerebellum, respectively. After controlling for global signal alone with the CONN approach (Pipeline 4), the left inferior frontal cluster shifted slightly inward into the sulcus (i.e., away from the extracranial activity and partial volume artefact) and additional clusters were again observed in the right cerebellum, the WM extending subcortically in the right parietal lobe, the left IPL (rostrodorsal), as well as in the right STG. Controlling for both residual head motion and CSF/edge effects (Pipeline 5) made the peak maxima shift to the right lateral orbital gyrus, with other clusters observed in the left IPL (2 clusters again), the right anterior superior temporal sulcus (antSTS), the right postcentral gyrus, and the right cerebellum. Note that the left IFG activation was still significant, but part of a more extensive cluster with a peak elsewhere (i.e., in the largest cluster peaking in the right lateral orbital gyrus). Adding GSR via the CONN single standardized scan-to-scan values (Pipeline 6) did not alter the cluster results compared to the previous pipeline except that the right postcentral gyrus cluster was now nonsignificant. After implementing GSR with LMGS (Pipeline 7), the maximum *Z*-score was observed in the left hemisphere but this time more posteriorly, in the left parietooccipital sulcus (POS), although the largest cluster was located in the left inferior frontal sulcus. Other clusters were observed in the antSTS, left IPL (caudal cluster), and right cerebellum. Finally, after controlling for all sources of noise with GSR as the LMGS method (Pipeline 8), the maximum *Z*-score was also located in left POS, with the left IFG again being the largest cluster, and other clusters were observed in the left and right cerebellum, left lateroventral fusiform gyrus, left IPL (caudal), and right STG (see Supplementary Table S2).

**Figure 5.**
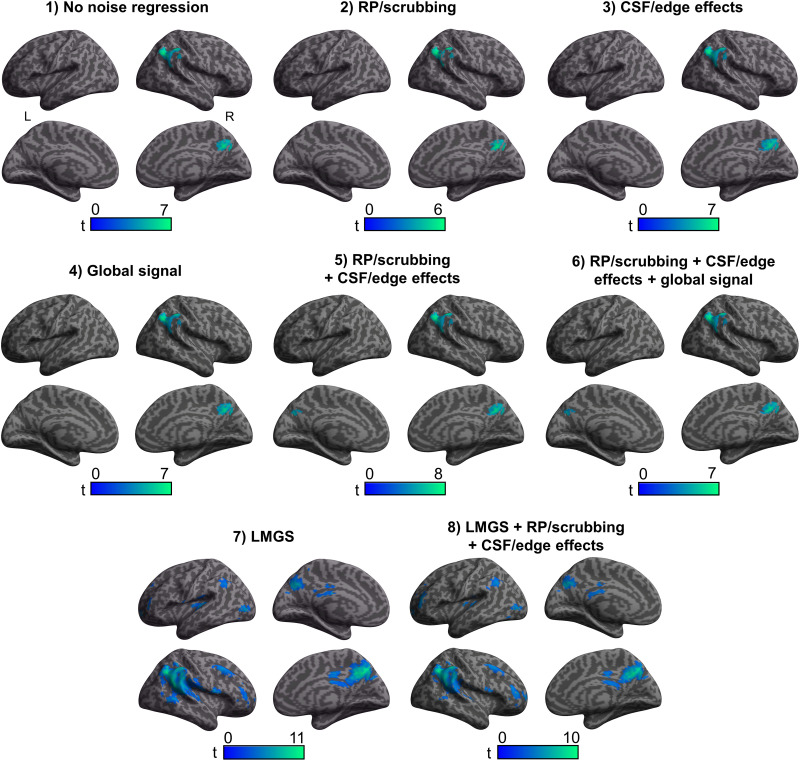
Cortical surface renderings showing significant BOLD signal changes for the contrast Definition < Control as a function of analysis pipeline. All results thresholded at *p* < 0.001 with a spatial extent cluster at *p* < 0.05 (FWE corrected).

For BOLD signal decreases (i.e., Definition < Control), results were comparable across Pipelines 1–6 (Table 2), with two clusters being consistently observed in the right rostroventral IPL and right precuneus (Figure 5 and Supplementary Table S3). However, the results significantly changed when using LMGS (Pipelines 7 and 8), manifesting as a greater number of significant clusters (from 2 to 9 in Pipeline 7 or 10 in Pipeline 8). For Pipeline 7, the first two peak clusters were identical to Pipelines 1–6 (right IPL and precuneus) but other clusters were also observed in the left inferior occipital gyrus, bilateral MFG (3 clusters), left cerebellum, left STG (primary auditory cortex), and rostrodorsal IPL. Pipeline 8 revealed the same clusters as Pipeline 7, with the addition of a cluster in the right IFG (Table S3). Using LMGS was associated with a larger peak cluster (about a fourfold increase in the number of voxels between Pipeline 1 and Pipelines 7 or 8). The MNI coordinates of the peak *Z*-score cluster remained similar across analysis pipelines but was slightly less lateral when using LMGS to control for regional variations in global signal intensity (Table 2).

**Table 2.** Number of significant BOLD signal clusters and characteristics of the peak *Z*-score cluster for the contrast Definition < Control as a function of noise removal analysis pipeline

Analysis pipeline	Number of significant clusters	Size of largest cluster	*Z* of largest cluster	Coordinates of peak at largest cluster			Peak location
1. No noise regression	2	1,239	5.02	56	−62	36	Right rostroventral IPL
2. RP/scrubbing	2	957	4.80	56	−62	36	Right rostroventral IPL
3. CSF/edge effects	2	1,630	5.33	54	−62	38	Right rostroventral IPL
4. CONN global signal	2	1,405	5.15	54	−62	38	Right rostroventral IPL
5. RP/scrubbing + CSF/edge effects	2	1,371	5.13	54	−64	38	Right rostroventral IPL
6. RP/scrubbing + CSF/edge effects + CONN global signal	2	1,454	5.16	54	−64	38	Right rostroventral IPL
7. LMGS	9	6,221	5.95	50	−60	38	Right rostroventral IPL
8. LMGS + RP/scrubbing + edge effects	10	4,011	5.74	52	−62	38	Right rostroventral IPL

*Note*. IPL = inferior parietal lobule, RP = realignment parameters, CSF = cerebrospinal fluid, CONN = CONN toolbox, LMGS = linear model of the global signal.

### Effects of Implementing GSR via LMGS on tSNR

**Figure 6.**
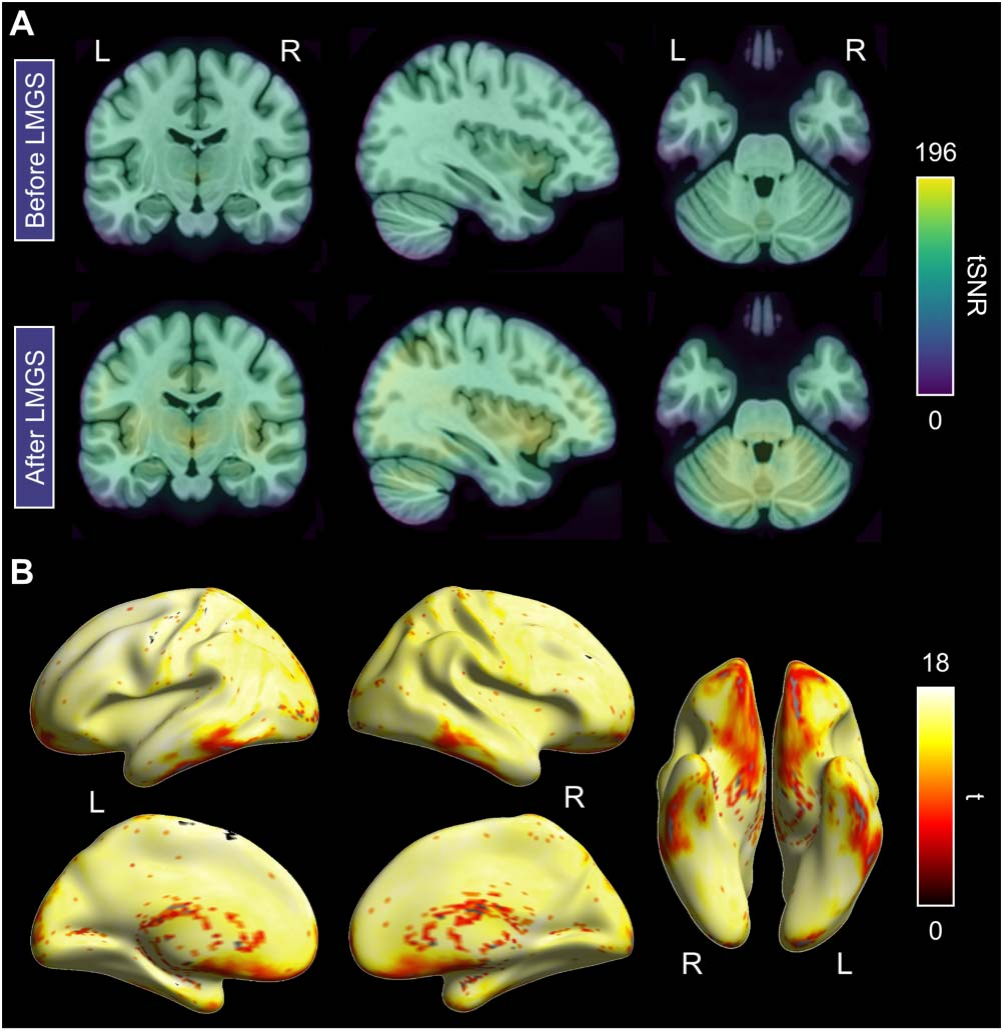
Changes in temporal signal-to-noise ratio (tSNR) between Pipelines 1 and 7. **A**. Maps showing the distribution and magnitude of tSNR for images following the application of global signal regression (GSR) with LMGS (Pipeline 7) compared to without (Pipeline 1), plotted on the MNI152 template in MRIcroGL. Scale is set at the maximum value across both maps. Slices are centred on MNI coordinates −36, −15, −30 in the ventral anterior temporal lobe. **B**. Regions showing significant tSNR increases for images with GSR applied via LMGS, rendered on an inflated cortical surface in SPM12. Height thresholded at *p* < 0.05 (FWE corrected) with spatial cluster extent at 5 for visualization purposes.

Another important point to consider when comparing analysis pipelines is whether they may significantly affect tSNR (Liu, 2016). As the LMGS GSR method resulted in different patterns of task-related spatial activation when added separately to Pipeline 5 (RP/scrubbing + CSF/edge effects) compared to adding the CONN global signal estimate from the masked data, which resulted in only minimal change, we investigated whether the former method affected tSNR. We found a significant and dramatic improvement in tSNR across all voxels in the brain mask following GSR with LMGS (Macey et al., 2004) that survived FWE correction at the whole brain level (Pipeline 1 vs. Pipeline 7; Figure 6A–B). Interestingly, this improvement was also statistically significant in brain regions with characteristically lower tSNR in fMRI, such as the ventral anterior temporal lobe (ATL; e.g., Hoffman & Lambon Ralph, 2018) and orbitofrontal cortex. To investigate the tSNR improvement in the ATL directly, we also computed the mean tSNR from two spherical regions of interest (ROIs) in the left and right ATL, respectively (6 mm radius at MNI coordinates: ±36, −15, −30; e.g., Binney et al., 2010; Rice et al., 2018). A paired *t* test showed significantly higher tSNR in both ROIs following GSR with LMGS, (*t*(17) = 14.845, *p* < 0.001, Cohen’s *d* = 3.5, and *t*(17) = 13.988, *p* < 0.001, *d* = 3.3, for the left and right ATL ROI, respectively).

## DISCUSSION

It is well known that overt speech produces artefacts in fMRI data that compromise the detection of task-related BOLD signal responses. We investigated the profiles of three different speech related noise components in MB fMRI: residual rigid body motion, CSF/edge, and global signal intensity fluctuations. We found that the variance associated with each source of production related noise in MB fMRI is statistically significant at FWE corrected thresholds and is spatially extensive. In addition, each noise source contributed unique variance when the others were statistically controlled. Overall, our findings show that applying a combined denoising approach tended to reduce and enhance production related BOLD signal increases and decreases, respectively. Implementing GSR with LMGS dramatically improved overall tSNR.

Although residual rigid body motion (realignment parameters and censored outlier volumes) contributed relatively little variance compared to the other noise sources we examined, it was nonetheless associated with significant clusters in several bilateral cortical regions. This result is consistent with earlier reports from studies using single band sequences and frame censoring with nonspeech tasks (e.g., Grootoonk et al., 2000; Siegel et al., 2014). Note that the number of censored volumes was relatively small in our sample (3.2% on average), with two thirds of the participants requiring only 10 or fewer volumes to be scrubbed. Correcting for residual motion at the individual participant level also had the least impact on the whole brain maximum *Z* value for task-related BOLD signal increases. However, it was the only denoising approach to diminish the maximum *Z* value for task-related decreases (Pipeline 2). While it is possible that using expansions of the number of realignment parameters from six in the present study to 24 might yield additional residual motion variance, findings from nonspeech tasks have been mixed (e.g., Jones et al., 2022) and the trade-off is a loss of degrees of freedom. Alternatively, a single mean framewise displacement (Power et al., 2014) value per volume could be adopted if researchers wish to minimize the loss of degrees of freedom in their analyses. Instead of using rigid body motion estimates, some MB EPI studies of speech production have included a regressor indexing the change in image intensity from one volume to the next (DVARS; e.g., Todorović et al., 2024). However, it is worth noting that changes in image intensity occur for various reasons in addition to head motion, such as respiration, cardiac cycles, and, importantly, task effects (Jones et al., 2022).

The application of component-based denoising approaches to both single echo and ME MB speech production fMRI data has increased in recent years (e.g., Janssen & Mendieta, 2020; Morales et al., 2022; Todorović et al., 2024). Here we used aCompCor’s PCA approach to estimate and remove the variance attributable to CSF/edge effects given their known association with changes in respiratory and cardiac cycles due to speech (e.g., Farthing et al., 2007; Mehta et al., 2006). This variance manifested as equally extensive signal increases and decreases, unlike the other two noise sources we investigated. While BOLD signal increases occurred in the expected CSF/edge spaces, the reductions were observed in bilateral frontal, temporal, and parietal lobe regions. Removing this variance diminished and enhanced both the whole brain maximum *Z* value and spatial extent of task-related BOLD signal increases and decreases, respectively (Pipeline 3). We note that, in theory, component-based denoising can be applied to an entire fMRI dataset allowing researchers to remove any components they might choose to designate as noise according to predetermined criteria (see Griffanti et al., 2017). By default, aCompCor also estimates and removes WM signals as nuisance regressors, and so MB fMRI studies of speech production using aCompCor have tended to adopt this default setting (e.g., Todorović et al., 2024). We did not, given the evidence that task-related BOLD responses in WM are likely to reflect effects of interest (Gawryluk et al., 2014; Grajauskas et al., 2019; Schilling et al., 2023).

The application of GSR in resting-state fMRI investigations has been much debated given the focus of those studies on the covariance in spontaneous low frequency fluctuations in BOLD signals (see Power, Laumann, et al., 2017). In fMRI studies of speech production, the task-related global signal fluctuations due to both respiration and extracranial muscle activity occurring during and following the speech envelope (e.g., Mehta et al., 2006) represent an unambiguous source of physiological noise when the goal is to investigate the various processes/mechanisms that precede articulation. Both sources of noise were apparent in the global signal variance calculated from our unmasked data and manifested primarily as BOLD signal increases over lateral frontal and anterior temporal lobe regions. Note that the use of brain masking in analyses does not mitigate the blurring of extracranial signals to voxels in these more lateral brain regions (i.e., partial volume artefacts). When we included the CONN estimate of average global signal intensity per scan as a nuisance regressor (Pipeline 4), this enhanced both the whole brain maximum *Z* value and spatial extent of task-related BOLD signal increases and decreases. Further, it moved the overall peak maxima for task-related BOLD signal increases in the left inferior frontal sulcus approximately 6 mm inward, that is, away from the extracranial noise source and partial volume artefacts. This was also the case when the GSR method that estimates regional variation in global signals (LMGS) was applied, which also resulted in a different, more posterior and medially located whole brain peak maximum (Macey et al., 2004), although it also reduced the spatial extent of task-related BOLD signal increases. As we noted in the Introduction, GSR is likely to be less useful when the goal is to study articulatory mechanisms during the speech envelope due to the increased likelihood of removing effects of interest. For the latter studies, an alternative strategy is to employ a sparse sampling acquisition with MB EPI (e.g., Correia et al., 2020).

Our findings also demonstrate that residual rigid body motion, CSF/edge, and global signal intensity fluctuations constitute independent/unique sources of noise in MB fMRI speech production data, as each source contributed significant variance even when the others were included in the design matrix as nuisance regressors. Accordingly, we investigated the effect of removing all three on our task-related BOLD signal responses (Pipelines 6 and 8). The net effect of removing all these sources of noise was to provide greater separation of regional activity as evidenced by an increased number of clusters for task-related BOLD signal increases with corresponding smaller spatial extents for both GSR approaches. However, we observed different results for the task-related activation maps depending on which GSR method was employed in combination with the residual motion and CSF/edge regressors. Specifically, adding the CONN regressor to the residual motion and CSF/edge regressors resulted in little differences in spatial activation (as compared to controlling for residual motion and CSF/edge regressors only; Pipeline 5), while adding the Macey et al. (2004) LMGS method resulted in more dramatic changes. The principal differences between the two GSR methods is that LMGS calculates global signal from unmasked data, removing the global signal in a separate step prior to analysis, whereas the CONN approach calculates the change in average BOLD signal within masked data, that is, excluding the extracranial noise sources, and includes it as a covariate of no-interest in the analysis. Therefore, it seems likely that much of the dramatic improvement in tSNR and changes in spatial activation we observed with LMGS can be attributed to its modelling and removal of the extracranial noise sources associated with spoken production.

### Limitations

We employed a task in which participants were required to delay their naming response to an auditorily presented sentence definition until a cue was presented. This delayed naming design temporally segregated the processes/mechanisms preceding and associated with the speech envelope. In conventional picture naming tasks, responses tend to be initiated between 400 and 600 ms post stimulus presentation (e.g., Indefrey & Levelt, 2004). It is possible that separation of global signals related to speech respiration and muscle activity using the same approach might be less efficient for more rapid responding, and so might result in more task-related BOLD signal being removed than desired. The contributions of all three noise sources are also likely to differ for MB fMRI studies of narrative production (e.g., Morales et al., 2022). For example, research has shown that inhalation is more likely to co-occur with syntactic boundaries than single words during narrative production, with the volume of air inhaled related to the length of utterance (Fuchs & Rochet-Capellan, 2021). Future MB fMRI studies using such designs might consider using a pneumatic belt to estimate and remove speech-related respiratory changes more precisely (e.g., Mehta et al., 2006).

In addition, as our fMRI data were acquired using a single echo MB EPI sequence (albeit dual-refocussed), our results are not necessarily generalizable to ME MB sequences. As we noted in the Introduction, ME MB sequences afford some advantages as well as disadvantages compared to single echo acquisitions. While component-based techniques have been proposed to be useful for separating TE-dependent non-BOLD artefacts from BOLD signals such as rigid body motion, Power, Plitt, Kundu, et al. (2017) showed that motion estimates calculated at different TEs in ME MB datasets were virtually identical. In addition, it is important to note that ME MB sequences still require that CSF/edge and global signals arising from respiration and muscle activity be estimated and removed as they are BOLD signals and the use of TRs > 1 s in MB EPI acquisitions will not allow adequate sampling of the latter noise sources (Power et al., 2018; Power, Plitt, Kundu, et al., 2017).

It is also well known that variations in spatial smoothing parameters can also affect the results of fMRI analyses (e.g., Hopfinger et al., 2000; Mikl et al., 2008). Here, we used an 8 mm FWHM Gaussian kernel for spatial smoothing, which is the default in SPM12 and so used most often in fMRI studies using this software, but we acknowledge that results with denoising approaches are likely to vary with different smoothing kernel sizes. Future studies could evaluate the impact of this variable on estimations of speech-related noise in MB EPI datasets.

While the primary goal of the present study was to characterize the different sources of speech-related noise and their respective influences on task-related activations in MB fMRI data, it should be noted that there is no gold-standard to evaluate the success of regression denoising methods for this purpose. Further, current methods are unlikely to account for all susceptibility artefacts (geometric distortions and signal dropouts) that occur dynamically during production (see also Box 19.1–2 in de Zubicaray & Piai, 2019). To date, the only fMRI acquisition sequence that has been shown to provide BOLD signal data free of production-related artefacts is sparse temporal sampling (e.g., Correia et al., 2020; Gracco et al., 2005). However, speech-related respiration signal changes will still affect image volumes from sparse acquisitions such that GSR might still be required. We recommend future studies compare speech-related noise in MB fMRI data acquired with sparse versus continuous sequences.

Finally, while our data are likely to be representative of most healthy adult participants performing a speech production task, rigid body motion is known to be exaggerated in paediatric and patient populations, so may require more aggressive denoising methods (e.g., Krishnamurthy et al., 2021; Yuan et al., 2009). In addition, the use of a single per-volume global signal value as a nuisance regressor is unlikely to be valid for patients with post-stroke aphasia as cerebral ischemia and hypoperfusion are known to result in regional variations in global signal intensity (e.g., Lv et al., 2013). Hence, LMGS GSR (Macey et al., 2004) may be preferable for removing global signal intensity fluctuations associated with speech production in these patients.

### Conclusion

Approaches to dealing with production-related artefacts have varied considerably in recent fMRI studies implementing continuous single band and MB EPI sequences to investigate pre-articulatory processes. Some have included only realignment parameters as nuisance regressors (e.g., Roos et al., 2023; Wolna et al., 2024), while others have denoised their time series using various components derived from ICA/PCA methods (e.g., Janssen & Mendieta, 2020; Morales et al., 2022; Todorović et al., 2024). We could find only one study that included (whole brain) global signal as a nuisance regressor (Todorović et al., 2024). This motivated us to investigate the impact of these noise sources on our production data, and methods for removing them. Our findings indicate that all three sources of noise make independent contributions, and removing their combined influence resulted in significant changes to task-related BOLD signal responses when compared to a pipeline involving only realigning and unwarping the data (e.g., Ekert et al., 2021). In our view, these changes in magnitude, location, and extent are sufficient to mandate the use of denoising strategies in speech production studies employing continuous MB EPI acquisitions. Note that our intent is not to be prescriptive about specific denoising pipelines as we acknowledge a combined approach such as the one we implemented is unlikely to be appropriate for all production experiments (e.g., speech motor control). We recommend production researchers further explore and report the outcomes of applying different denoising approaches to MB fMRI data acquired across various experimental designs.

## ACKNOWLEDGMENTS

The authors would like to thank the editor and two anonymous reviewers for their helpful comments on previous versions of the manuscript.

## FUNDING INFORMATION

Greig I. de Zubicaray, Australian Research Council (https://dx.doi.org/10.13039/501100000923), Award ID: DP200100127.

## AUTHOR CONTRIBUTIONS

**Angelique Volfart**: Conceptualization; Data curation; Formal analysis; Investigation; Methodology; Project administration; Software; Visualization; Writing – original draft; Writing – review & editing. **Katie L. McMahon**: Conceptualization; Funding acquisition; Methodology; Software; Supervision; Writing – original draft; Writing – review & editing. **Greig I. de Zubicaray**: Conceptualization; Formal analysis; Funding acquisition; Methodology; Supervision; Writing – original draft; Writing – review & editing.

## DATA AND CODE AVAILABILITY STATEMENTS

Neuroimaging data are available in an OpenNeuro repository (doi:10.18112/openneuro.ds004957.v1.0.0). Stimuli and PsychoPy experiment script are available in an OSF repository (https://osf.io/fh5xb/?view_only=de0a5006046b4003b32e26eb501a3dfe).

## Supplementary Material



## TECHNICAL TERMS

Rigid body motion: Refers to a simplified form of movement, either shifting in *x*, *y*, *z*, or translating or rotating in one of three directions.
Partial voluming artefacts: Occur when different tissue types (e.g., grey matter and CSF) are present in the same voxel.
Multiband EPI: Also, *simultaneous multislice* or *multiband fMRI acquisition*; involves imaging multiple brain slices simultaneously, resulting in faster imaging time.
Acceleration factor: In a multiband sequence, indicates how many slices are excited simultaneously.
