# Supplementary material for: A Comparison of Denoising Approaches for Spoken Word Production Related Artefacts in Continuous Multiband fMRI Data
Source: Neurobiol Lang (Camb). 2024 Sep 11;5(4):901–21. doi: 10.1162/nol_a_00151 (PMC11410355; doi:10.1162/nol_a_00151)
Supplement: Supplementary file 1 [file nol-5-4-901-s001.pdf]

Supplementary Material

A comparison of denoising approaches for spoken word production related artefacts in continuous multiband fMRI data

Supplementary tables

Table 1. Cerebral regions showing significant BOLD increases and decreases for each individual source of noise when controlling for others (unique variance; data from pipeline 5).

	Peak MNI (x y z)			Z-score	Cluster size (N voxels)
Residual head motion > Baseline					
Left postcentral gyrus (trunk area)	-18	-42	68	5.20	943
Left precentral gyrus (head/face area)	-46	-8	48	4.77	224
Right SPL	10	-58	62	4.68	246
Left caudal lingual gyrus	-10	-80	-10	4.30	219
Left lateral superior occipital gyrus	-24	-78	34	3.80	229
Residual head motion < Baseline					
Right ventral MFG	48	42	22	5.91	1167
Right dorsolateral SFG	16	2	64	4.65	156
Right dorsolateral putamen	36	-12	-2	4.50	367
Right caudal middle temporal gyrus	66	-20	-16	4.17	167
Right medial SFG	10	26	58	4.07	233
CSF/edge effects > Baseline					
Ventral cingulate gyrus	0	-48	6	6.76	23339
Medial SFG	0	10	70	5.53	956
Left lateral MFG	-36	62	-2	4.48	174
Right MFG	30	64	10	4.17	166
CSF/edge effects < Baseline					
Right caudal ventrolateral precentral gyrus	46	2	34	6.66	41486
Right precentral gyrus (trunk area)	12	-24	70	5.95	537
Right rostroventral fusiform gyrus	40	-6	-34	4.24	545

Brain stem	-2	-24	-34	4.21	282
Global signal changes > Baseline					
Right medial SFG	8	20	62	5.47	7364
Right thalamus	2	-18	-6	5.44	2413
Left rostral superior temporal gyrus	-50	0	-10	4.98	2435
Left orbital gyrus	-2	32	-14	4.35	324
Brain stem	10	-38	-38	4.31	461
Right caudal IFG	56	26	22	4.30	885
Right medial SFG	2	58	16	4.15	294
Left caudal cingulate gyrus	-8	-18	40	4.14	219
Right caudal IPL	64	-48	26	4.09	139
Right caudal dorsolateral precentral gyrus	30	-16	58	4.08	144
Left caudal IPL	-58	-44	22	4.06	216
Left caudal IPL	-56	-44	46	4.05	275
Right rostroventral IPL	60	-22	40	4.01	509
Right caudodorsal cingulate gyrus	2	14	36	3.99	152
Left rostral SPL	-22	-62	64	3.92	109
Right opercular IFG	42	34	4	3.84	120
Left dorsal caudate nucleus	-4	4	8	3.77	190
Left pregenual cingulate gyrus	-8	32	24	3.51	120
Global signal changes < Baseline					
None					

Note: Mask value at 80%, height threshold at $p < .001$ and spatial cluster extent at $p < .05$. Peak locations were determined using Brainnetome atlas. IFG=inferior frontal gyrus; IPL=inferior parietal lobule; MFG=middle frontal gyrus; SFG=superior frontal gyrus; SPL=superior parietal lobule.

Table S2. Cerebral regions showing significant BOLD changes for the contrast Definition > Control as a function of the noise removal analysis pipeline.

	Peak MNI (x y z)			Z-score	Cluster size (N voxels)
Pipeline 1 – No noise regression					
Left rostral IFG	-50	28	2	6.26	25423
Right lobule X of cerebellum	22	-36	-42	5.28	369
White matter of the right parietal lobe	32	-30	30	5.11	312
Left rostrodorsal IPL	-40	-36	38	4.74	339
Left caudal IPL	-32	-70	28	4.59	1243
Pipeline 2 – RP/scrubbing					
Left rostral IFG	-50	28	2	6.27	35939
Left rostrodorsal IPL	-40	-36	38	5.42	423
Right lobule X of cerebellum	22	-38	-38	5.26	301
Left caudal IPL	-32	-70	28	4.88	1444
Right lateral STG	56	12	-16	4.84	563
Pipeline 3 – CSF/edge effects					
Left rostral IFG	-50	28	2	5.99	17607
Left rostral STG	-58	-8	-8	5.54	909
Left caudal IPL	-32	-70	28	4.58	891
Right lobule VI of cerebellum	16	-70	-28	4.00	827
Pipeline 4 – CONN global signal					
Left inferior frontal sulcus	-44	32	6	6.40	28043
Right lobule X of cerebellum	22	-36	-42	5.21	394
Left rostrodorsal IPL	-40	-36	38	4.69	291
Right lateral STG	54	14	-20	4.51	261
White matter of the right parietal lobe	30	-30	28	4.49	294
Pipeline 5 - RP/scrubbing + CSF/ edge effects					
Right lateral orbital gyrus	30	28	-2	6.14	24564
Left rostrodorsal IPL	-40	-36	38	5.47	370
Left caudal IPL	-32	-70	28	4.92	991
Right lobule VIII of cerebellum	30	-40	-44	4.78	219
Right anterior superior temporal sulcus	52	-8	-18	4.70	465
Right Crus I of cerebellum	42	-66	-26	4.54	1643
Lobule IX of vermis	0	-52	-36	4.22	276

Right postcentral gyrus (upper limb area)	48	-6	42	4.07	222
Pipeline 6 - RP/scrubbing + CSF/ edge effects + CONN global signal					
Right lateral orbital gyrus	30	26	-2	6.11	23735
Left rostrodorsal IPL	-40	-36	38	5.47	355
Left caudal IPL	-32	-70	28	4.91	1002
Right lobule VIII of cerebellum	30	-40	-44	4.83	225
Right Crus I of cerebellum	42	-66	-26	4.65	1630
Right anterior superior temporal sulcus	52	-8	-18	4.54	385
Lobule IX of vermis	0	-52	-36	4.26	271
Pipeline 7 – LMGS					
Left parietooccipital sulcus	-6	-60	6	6.29	4033
Left inferior frontal sulcus	-44	32	8	6.22	11543
Left anterior superior temporal sulcus	-52	-6	-18	5.98	851
Right lobule VI of cerebellum	34	-40	-42	5.14	243
Left caudal IPL	-32	-74	36	4.92	744
Right lobule VI of cerebellum	32	-60	-26	4.50	568
Pipeline 8 – LMGS + RP/scrubbing + CSF/ edge effects					
Left parietooccipital sulcus	-6	-58	8	6.28	2405
Left rostral IFG	-48	28	0	6.15	13047
Right lobule VI of cerebellum	32	-60	-26	5.07	743
Left lateroventral fusiform gyrus	-46	-48	-20	4.87	1900
Left caudal IPL	-34	-72	30	4.81	653
Right lateral STG	54	10	-14	4.66	191
Lobule IX of vermis	2	-54	-32	4.36	170

Note: Height threshold at $p < .001$ and spatial cluster extent at $p < .05$. Peak locations were determined using Brainnetome atlas, except for peak clusters in the cerebellum that were located using aal3 atlas. IFG=inferior frontal gyrus; IPL=inferior parietal lobule; STG=superior temporal gyrus.

Table S3. Cerebral regions showing significant BOLD changes for the contrast Definition < Control as a function of the noise removal analysis pipeline.

	Peak MNI (x y z)			Z-score	Cluster size (N voxels)
Pipeline 1 – No noise regression					
Right rostroventral IPL	56	-62	36	5.02	1239
Right precuneus (Lc1)	10	-58	34	4.54	476
Pipeline 2 – RP/scrubbing					
Right rostroventral IPL	56	-62	36	4.80	957
Right precuneus (Lc1)	10	-58	34	4.51	368
Pipeline 3 – CSF/edge effects					
Right rostroventral IPL	54	-62	38	5.33	1630
Right precuneus (Lc1)	10	-58	34	4.61	639
Pipeline 4 – CONN global signal					
Right rostroventral IPL	54	-62	38	5.15	1405
Right precuneus (Lc1)	10	-62	36	4.62	525
Pipeline 5 - RP/scrubbing + CSF/ edge effects					
Right rostroventral IPL	54	-64	38	5.13	1371
Right precuneus (Lc1)	10	-58	34	4.65	627
Pipeline 6 - RP/scrubbing + CSF/ edge effects + CONN global signal					
Right rostroventral IPL	54	-64	38	5.16	1454
Right precuneus (Lc1)	10	-58	34	4.74	654
Pipeline 7 – LMGS					
Right rostroventral IPL	50	-60	38	5.95	6221
Right precuneus (Lc1)	8	-60	36	5.83	2929
Left inferior occipital gyrus	-46	-84	-4	5.17	268
Left ventral MFG	-42	54	8	5.12	590
Left Crus II of cerebellum	-34	-76	-40	4.95	208
Left STG (TE1.0 and TE1.2)	-40	-24	8	4.78	637
Right ventrolateral MFG	32	18	46	4.58	815
Right ventral MFG	44	54	8	4.54	438
Left rostrodorsal IPL	-42	-58	48	4.33	403
Pipeline 8 – LMGS + RP/scrubbing + CSF/ edge effects					
Right rostroventral IPL	52	-62	38	5.74	4011
Right precuneus (Lc1)	10	-58	34	5.52	2432

Left MFG	-32	62	16	5.10	907
Right ventrolateral MFG	32	18	46	4.74	667
Left inferior occipital gyrus	-46	-84	-4	4.65	207
Left STG (TE1.0 and TE1.2)	-46	-18	8	4.61	378
Left Crus II of cerebellum	-36	-76	-40	4.52	212
Right ventral IFG	54	10	10	4.46	159
Right MFG	26	62	6	4.41	675
Left rostrodorsal IPL	-44	-62	50	4.27	430

Note: Height threshold at $p < .001$ and spatial cluster extent at $p < .05$. Peak locations were determined using Brainnetome atlas, except for peak clusters in the cerebellum that were located using aal3 atlas. IFG=inferior frontal gyrus; IPL=inferior parietal lobule; MFG=middle frontal gyrus; STG=superior temporal gyrus.
